# Anger and Anxiety as Sequential Predictors of Fatigue: A Two‐Wave Longitudinal Study

**DOI:** 10.1155/da/8772749

**Published:** 2026-02-23

**Authors:** Shegang Zhou, Haoran Guo, Xiaofu Liu, Lulu Ding, Rong Fu, Yanan Nie, Lingling Wang

**Affiliations:** ^1^ Department of Education, Henan Normal University, Xinxiang, China, henannu.edu.cn; ^2^ Mental Health Center, Henan Normal University, Xinxiang, China, henannu.edu.cn; ^3^ Division of Textbook Management, Henan Provincial Department of Education, Zhengzhou, China

**Keywords:** anger, anxiety, college mental health, fatigue, longitudinal predictive pathway

## Abstract

**Background:**

Fatigue occurs at a high rate among college students. Anger and anxiety are thought to be important triggers for fatigue. The mechanisms underlying the association between anger and fatigue remain unclear. The objective of this longitudinal study was to examine a sequential predictive pathway in which anger prospectively predicts fatigue through anxiety among Chinese university students.

**Method:**

The participants in this current study were 3475 college students (2686 females and 789 males, 15–42 years old, *M* = 19.78 years, SD = 1.96) from a university in Xinxiang City, Henan province, mainland China. Data for this two‐wave longitudinal study were collected via online surveys at two timepoints: October 2023 (T1) and April 2024 (T2). The Patient‐Reported Outcomes Measurement Information System (PROMIS) Anger scale, Anxiety scale, and Fatigue scale were used to assess state‐like emotional functioning (anger and anxiety assessed over the past 7 days) at each wave. These week‐based assessments were conceptualized as indicators of participants’ proximal emotional conditions at each timepoint rather than emotions persisting across the entire 6‐month interval.

**Results:**

In longitudinal structural equation models, T1 anger significantly predicted higher anxiety at follow‐up (*β* = 0.314, 95% CI [0.267,0.362], *p* < 0.001). The autoregressive effect of T1 anxiety on T2 anxiety was also significant (*β* = 0.315, 95% CI [0.264, 0.368], *p* < 0.001). Likewise, T1 anxiety significantly predicted increases in T2 fatigue (*β* = 0.267, 95% CI [0.225,0.310], *p* < 0.001) even after accounting for the autoregressive effect of T1 fatigue (*β* = 0.339, 95% CI [0.296,0.382], *p* < 0.001). To enhance transparency, a supplementary structural model was estimated to assess the direct predictive effect of T1 anger on T2 fatigue. After controlling for T1 fatigue, T1 anger showed a significant direct association with T2 fatigue (*β* = 0.396, 95% CI [0.316,0.477], *p* < 0.001). Overall, the findings support a sequential prediction pattern in which earlier anger predicts later increases in anxiety, which subsequently predict increases in fatigue.

**Conclusions:**

This study provides evidence for a sequential predictive pathway linking anger to fatigue via anxiety in college students. These findings suggest that baseline state‐like emotional functioning—rather than enduring traits—can initiate longer‐term psychological processes. Interventions that reduce anger and anxiety at their point of emergence may help interrupt this temporal chain and ultimately alleviate fatigue.

## 1. Introduction

Fatigue is a state of persistent physical and mental exhaustion that cannot be relieved by rest and often impairs daily functioning [1]. In contemporary society, mounting academic and social pressures have rendered fatigue a widespread concern among college populations [2]. A study of 864 Chinese undergraduates showed that ~40% self‐rated as highly fatigued [3]. Luo et al. [4] found that in a large survey of university students, the prevalence of chronic fatigue syndrome (CFS) was 6.25%, and fatigue symptoms were frequently reported, indicating that both general fatigue and CFS are nonnegligible among college populations. Those who rated themselves as fatigued were more vulnerable to sleep disturbances, declines in physical and mental health, and poorer academic performance [3–6]. Thus, clarifying factors that contribute to fatigue and identifying the psychological mechanisms linking negative emotions and fatigue is essential for developing targeted interventions.

Fatigue arises from a constellation of stress‐related, physiological, and psychological factors. Stress exposure—such as heavy academic demands or illness—can directly precipitate fatigue [7], while intrinsic physiological states, including poor sleep and reduced motivation, contribute to its persistence [5]. Increasingly, psychological determinants—especially negative emotions—are recognized as central drivers of fatigue [8]. The cognitive‐affective stress model suggests that stress‐induced negative affect consumes cognitive and emotional resources, which in turn promotes psychological fatigue and subsequent physical exhaustion [9]. In student populations, negative affect has been shown to significantly predict fatigue severity [10].

Anger arises when goal pursuit is blocked or expectations are violated and is conceptualized as a high‐arousal negative emotion [11]. According to self‐regulation theory, sustained anger requires ongoing regulatory effort to inhibit impulses and manage emotional responses, thereby increasing cognitive load and depleting self‐regulatory resources [12, 13]. Consistent with this framework, empirical studies show that prolonged anger is associated with heightened sympathetic activation, elevated stress hormones (e.g., cortisol) [14, 15], and greater reliance on effortful regulation strategies such as suppression and rumination [16], all of which have been linked to increased fatigue [17, 18]. Conservation of Resources (COR) theory further provides a coherent framework for understanding these processes. COR posits that individuals strive to retain, protect, and build resources, while stress results from resource loss or inadequate resource gain [19]. Anger, especially when recurring, consumes cognitive, emotional, and social resources through heightened vigilance, conflict, and regulation demands. Over time, such resource depletion culminates in chronic fatigue. Empirically, anger is positively associated with fatigue across postpartum, pain, and neurological populations [18, 20, 21]. Some studies suggest anger may operate as an intermediary linking stress exposure to fatigue [22], although the mechanisms remain underexamined.

Anxiety, characterized by anticipatory worry and heightened cognitive arousal, has been consistently associated with fatigue [23–26]. However, longitudinal findings regarding the direction of this association remain inconclusive: some studies suggest that fatigue may precede or exacerbate anxiety symptoms [27, 28], whereas others provide evidence that anxiety prospectively predicts subsequent fatigue [24, 29–31]. Anxiety sensitivity and persistent threat appraisal intensify physiological arousal and reduce perceived control, contributing to sustained energy depletion [30, 32]. Although the association between anxiety and fatigue has been extensively documented in clinical populations, comparatively less attention has been devoted to examining these pathways among college students.

Anxiety and anger are both prevalent negative emotions, yet they represent conceptually and functionally distinct affective states that are often conflated in the literature [33, 34]. Conceptually, anxiety reflects anticipatory worry about potential failure, whereas anger arises when goals are blocked or actions are thwarted [11]. Neurobiologically, although both emotions can be initiated by threat‐related fight‐or‐flight responses [35], the autonomic pathways and neural circuits underlying anger and anxiety are distinguishable [36]. Functionally, anxiety typically motivates avoidance behavior, whereas anger is associated with approach‐oriented tendencies [37]. Together, these distinctions underscore that anxiety and anger, despite their frequent co‐occurrence, constitute separable emotional constructs.

Importantly, recent theoretical and empirical work suggests a sequential relationship, in which anger may precipitate anxiety. Anger activates maladaptive schemas (e.g., vulnerability and abandonment), heightening threat monitoring and catastrophic thinking, which in turn increase anxiety [38–40]. Longitudinal evidence shows that elevated anger predicts subsequent increases in anxiety [41], supporting a plausible temporal chain.

Integrating these perspectives, anger may erode regulatory resources and deplete emotional and cognitive reserves, generating heightened vigilance, rumination, and perceived threat—key cognitive features of anxiety. Building on this theoretical foundation, prior research suggests that anger is associated with maladaptive emotion‐regulation responses, such as expressive suppression, experiential avoidance, and anger‐related rumination, which prolong negative affect and increase cognitive load [42, 43]. These regulatory patterns not only sustain anger activation but also amplify threat sensitivity, thereby increasing vulnerability to subsequent anxiety responses. In turn, anxiety maintains chronic hyperarousal, avoidance behaviors, and inefficient emotion‐regulation strategies, all of which contribute to fatigue through continuous resource consumption. Furthermore, anxiety‐related attentional biases toward threat, rumination, and behavioral avoidance have been shown to restrict opportunities for physiological and psychological recovery, reinforcing a sustained state of cognitive overactivation that accelerates the development of fatigue [32].

In summary, existing research consistently demonstrates close interrelationships among anger, anxiety, and fatigue, with increasing empirical evidence supporting these associations. However, despite these advances, the precise mechanisms by which they interact remain poorly understood. This study therefore adopts a longitudinal design to examine whether the hypothesized relationships among these variables hold empirically. Specifically, we hypothesized that anger is positively associated with anxiety and that anger at time point T1 can predict anxiety at time point T2 (Hypothesis 1). Additionally, we hypothesized that anxiety and fatigue are significantly positively correlated and that anxiety at time point T1 can significantly predict fatigue at time point T2 (Hypothesis 2). Finally, we hypothesized a sequential predictive chain, such that T1 anger would forecast increases in T2 anxiety, which would subsequently contribute to elevated fatigue (Hypothesis 3).

## 2. Materials and Methods

### 2.1. Samples and Procedures

Data for this analysis were derived from a longitudinal questionnaire study of undergraduate students at a university in Henan Province, China. Using cluster sampling, we administered surveys at baseline (T1: October 2023) and follow‐up (T2: April 2024), with a 6‐month interval. Prior to participation, trained research assistants fluent in the local dialect provided detailed oral explanations about the study’s purpose, procedures, risks, benefits, and confidentiality measures. Participants were informed in Mandarin to ensure cultural and linguistic appropriateness. Verbal consent was explicitly obtained from all participants only after confirmation of their understanding. This process was documented via completion of a Chinese electronic checklist, which recorded receipt of study information and voluntary agreement. Then, all participants completed the assessment, which was set up on a professional online survey platform named Wen Juan Xing, using their mobile phones. This study was approved by the Ethics Committee of the Psychology Department at Henan Normal University (Project Identification Code 2022100003). All participants were advised of their voluntary participation rights and ability to withdraw without consequence.

At T1, we applied several predefined data‐quality screening procedures to identify and remove invalid questionnaires. First, responses completed in less than 120 s were excluded, as such durations fall below the minimum time needed to attentively read and complete all items on the survey. Second, questionnaires with more than 10% missing items on any scale were removed. Third, we screened for straightlining by excluding cases in which participants selected the same response option for more than 80% of the items. In addition, two instructional attention‐check items were included (e.g., “Please select ‘Strongly Agree.’”). Participants who failed either item were excluded. After applying these criteria, a total of 3679 undergraduates comprised the valid baseline sample at T1. At T2 (6‐month follow‐up), the same data‐quality screening procedures were applied, and retention was 94.46% (*n* = 3475) after excluding incomplete or invalid responses. The final analytical sample consisted of 3475 undergraduates (2686 females; 789 males; age range 15–42 years, *M* = 19.78, SD = 1.96), as detailed in Table 1.

**Table 1:** Demographic characteristics of participants at baseline (*N* = 3475).

Sociodemographic variables	*N*	Percent
Sex		
Men	789	22.7%
Female	2686	77.3%
Age		
15–18	893	25.7%
18–25	2533	72.9%
>25	49	1.4%
Education level		
Undergraduate student	3218	92.6%
Postgraduate student or higher	257	7.4%

*Note*: “Undergraduate student” refers to participants currently enrolled in bachelor‐level programs. “Postgraduate student or above” refers to those enrolled in master’s or doctoral programs.

### 2.2. Measures

#### 2.2.1. Fatigue

The Patient‐Reported Outcomes Measurement Information System (PROMIS) Short Form v3.0‐Fatigue consists of 10 items assessing perceived fatigue. It captures symptoms ranging from mild tiredness to debilitating exhaustion that interferes with daily functioning. The short form assesses fatigue over the past 7 days. Example items include: “Being tired made it hard for me to keep up with my schoolwork,” “Being tired made it hard for me to play or go out with my friends as much as I’d like,” and “I felt weak.” Items are rated on a 5‐point scale from 1 (Never) to 5 (Almost always), with higher scores indicating greater fatigue severity. In the current study, PROMIS fatigue scores were conceptualized as indicators of participants’ state‐like fatigue levels at each wave—a proximal emotional/physical condition that may initiate longer‐term processes—rather than reflecting stable trait fatigue or implying 6‐month continuity. Cronbach’s alpha values at T1 and T2 were 0.96 and 0.97, respectively.

#### 2.2.2. Anger

The PROMIS Short Form v1.1‐Anger includes 5 items assessing perceived anger. Items are rated from 1 (Never) to 5 (Almost always) and include example items such as “I felt fed up,” “I felt mad,” and “I was so angry I felt like yelling at somebody.” Higher scores reflect greater anger. Following prior longitudinal research, PROMIS anger scores were conceptualized as state‐like emotional functioning at each wave. Instead, we followed prior longitudinal work in conceptualizing PROMIS anger scores as state‐like emotional functioning at each timepoint—that is, a short‐term emotional condition that may initiate downstream affective or behavioral processes over the following months. In the current study, the Cronbach’s alpha values for the sample were 0.89 (T1) and 0.90 (T2).

#### 2.2.3. Anxiety

The PROMIS Short Form v1.0‐Anxiety consists of 8 items, assessing perceived anxiety (e.g., “I felt fearful,” “I found it hard to focus on anything other than my anxiety,” and “My worries overwhelmed me”). Responses range from 1 (Never) to 5 (Almost always), with higher scores indicating more severe anxiety. As with anger and fatigue, PROMIS anxiety scores represent participants’ current anxiety levels at each wave, based on a 1‐week recall window. These scores provide time‐anchored indices of anxiety that may forecast later changes, rather than reflecting enduring trait anxiety or assuming emotional continuity over 6 months. Cronbach’s alpha values for the sample at T1 and T2 were both 0.95.

### 2.3. Longitudinal Measurement Invariance

In this study we examined four types of measurement invariance: configural, metric, scalar, and strict invariance [44–46]. Following established methodological recommendations for invariance testing, decisions regarding measurement equivalence were based primarily on changes in model fit indices rather than their absolute values. Specifically, changes in CFI (ΔCFI ≤0.01) and RMSEA (ΔRMSEA ≤0.015) were used as the main criteria for evaluating invariance across models [47]. When fit indices yielded discrepant indications, greater weight was placed on changes in CFI, as RMSEA is known to be sensitive to model constraints and degrees of freedom, particularly in longitudinal invariance models with large samples. The results of the invariance tests are presented in Table 2, together with chi‐square statistics. Because chi‐square tests are highly sensitive to sample size—as in the present study—these values were interpreted cautiously and were not used as the primary basis for invariance decisions [48].

**Table 2:** Model fit indices of nested longitudinal invariance models (*N* = 3475).

Variable	Model	*χ* ^2^	df	RMSEA	CFI	TLI	SRMR	Model comparison	Δ*χ* ^2^	Δdf	*p*	ΔCFI	ΔRMSEA
Anger	M1: configural invariance	4304.787	29	0.206	0.854	0.773	0.076						
	M2: metric invariance	4338.154	32	0.197	0.853	0.793	0.077	M2–M1	33.367	3	<0.05	0.001	0.009
	M3: scalar invariance	4420.534	37	0.185	0.850	0.818	0.078	M3–M2	82.380	5	<0.05	0.003	0.001
	M4: strict invariance	4516.634	42	0.175	0.847	0.836	0.081	M4–M3	96.100	5	<0.05	0.003	0.003
Anxiety	M1: configural invariance	2105.354	95	0.078	0.963	0.953	0.020						
	M2: metric invariance	2109.064	101	0.076	0.963	0.956	0.020	M2–M1	3.510	6	<0.05	0.000	0.002
	M3: scalar invariance	2128.861	109	0.073	0.962	0.959	0.020	M3–M2	19.797	8	<0.05	0.001	0.003
	M4: strict invariance	2138.951	117	0.071	0.962	0.961	0.021	M4–M3	10.090	8	<0.05	0.000	0.002
Fatigue	M1: configural invariance	6106.580	159	0.104	0.926	0.911	0.030						
	M2: metric invariance	6121.346	167	0.101	0.926	0.916	0.031	M2–M1	14.766	8	<0.05	0.000	0.003
	M3: scalar invariance	6228.165	177	0.099	0.925	0.919	0.031	M3–M2	106.819	10	<0.05	0.001	0.002
	M4: strict invariance	6650.418	187	0.100	0.919	0.918	0.036	M4–M3	422.253	10	<0.05	0.006	0.005

*Note:* M1 to M4 represent configural invariance, metric invariance, scalar invariance, and strict invariance models, respectively. *p* indicates statistical significance. Although the absolute RMSEA values were relatively high across invariance models, changes in CFI (ΔCFI ≤0.01) and RMSEA (ΔRMSEA ≤0.015) met commonly recommended criteria for longitudinal measurement invariance [47, 48]; RMSEA is known to be sensitive to model constraints and degrees of freedom in longitudinal invariance testing and should therefore be interpreted with caution.

Abbreviations: Δ, change. *χ^2^*, chi‐square. CFI, comparative fit index. df, degrees of freedom. RMSEA, root mean square error of approximation. SRMR, standardized root mean square. TLI, Tucker–Lewis index.

### 2.4. Data Analysis

All statistical analyses were performed using SPSS 25.0 and Amos 25.0. Descriptive statistics and Pearson correlations among the main study variables were first computed in SPSS. To evaluate the theoretically ordered longitudinal predictive relationships among anger, anxiety, and fatigue, we specified a two‐wave autoregressive structural equation model in AMOS 25.0, rather than applying a traditional mediation framework. Following the proposed temporal sequence, T1 anger was modeled as a predictor of T2 anxiety while controlling for T1 anxiety, and T1 anxiety was modeled as a predictor of T2 fatigue while controlling for T1 anger and T1 fatigue. Gender was included as a covariate to account for potential demographic influences. Controlling for baseline levels of each outcome allowed us to estimate change over time and isolate the unique predictive effects consistent with the theoretical temporal ordering. Missing data between T1 and T2 were handled using Full Information Maximum Likelihood (FIML) in AMOS. FIML incorporates all available information under the missing‐at‐random (MAR) assumption and is widely recommended for longitudinal SEM because it minimizes bias and preserves statistical power. Attrition analyses indicated that participants who did not complete T2 did not differ significantly from retained participants on baseline anger, anxiety, or fatigue, supporting the plausibility of the MAR assumption. No listwise deletion or mean imputation procedures were used; all SEM estimates were based on FIML. Model fit was evaluated using multiple indices, with Comparative Fit Index (CFI) >0.95, Tucker–Lewis Index (TLI) >0.95, Root Mean Square Error of Approximation (RMSEA) <0.08, and Standardized Root Mean Square Residual (SRMR) <0.08 considered indicative of good fit [49]. We also note that in two‐wave autoregressive models, RMSEA can appear artificially elevated due to the model’s limited structural complexity and the penalty RMSEA imposes on models with relatively few freely estimated residual covariances—even when other global fit indices perform well [50]. Accordingly, CFI and TLI were treated as the primary indicators for evaluating overall model adequacy. To assess potential common method variance due to the use of self‐report scales administered within a single survey session, we conducted both Harman’s single‐factor test and confirmatory factor analysis comparisons. The results indicated that no single factor accounted for the majority of variance, and the hypothesized three‐factor model demonstrated substantially superior fit relative to a single‐factor model, suggesting that CMV is unlikely to pose a major threat to construct validity.

## 3. Results

### 3.1. Sample Demographics

Descriptive statistics and bivariate correlations for anger, anxiety, and fatigue are presented in Tables 3 and 4 (T1 = baseline assessment; T2 = follow‐up assessment 6 months later). Pearson correlation analyses indicated significant associations among anger, anxiety, and fatigue at both time points. In addition to correlations, paired‐samples *t*‐tests were used to compare mean levels of anger, anxiety, and fatigue between T1 and T2. All three constructs showed small but statistically significant decreases over time (*p* < 0.001; see Table S1), consistent with modest improvement in students’ emotional states across the 6‐month interval.

**Table 3:** Descriptive statistics among primary study variables (*N* = 3475).

Variable	Mean	SD	Min	Max
Age	19.78	1.97	15	42
T1 anger	10.66	3.62	5	22
T1 anxiety	16.49	6.19	8	35
T1 fatigue	21.81	7.69	10	46
T2 anger	9.92	3.78	5	21
T2 anxiety	15.59	6.02	8	34
T2 fatigue	20.18	7.91	9	44

*Note:* SD = standard deviation; Min = minimum value; Max = maximum value.

**Table 4:** Bivariate correlations among primary study variables (*N* = 3475).

Variable	1	2	3	4	5	6
1.T1 anger	1					
2.T1 anxiety	0.51 ^∗∗^	1				
3.T1 fatigue	0.53 ^∗∗^	0.58 ^∗∗^	1			
4.T2 anger	0.45 ^∗∗^	0.43 ^∗∗^	0.41 ^∗∗^	1		
5.T2 anxiety	0.42 ^∗∗^	0.48 ^∗∗^	0.43 ^∗∗^	0.66 ^∗∗^	1	
6.T2 fatigue	0.40 ^∗∗^	0.44 ^∗∗^	0.48 ^∗∗^	0.62 ^∗∗^	0.62 ^∗∗^	1

*Note:* The table presents correlation coefficients between variables. T1 = baseline assessment; T2 = follow‐up assessment 6 months later.

^∗∗^ *p* < 0.01.

### 3.2. Psychometric Properties

To evaluate the reliability and convergent validity of the latent constructs, composite reliability (CR) and average variance extracted (AVE) were calculated using standardized factor loadings from the confirmatory factor models. Across all constructs and time points, CR values ranged from 0.89 to 0.97, exceeding the recommended criterion of 0.70, and AVE values ranged from 0.63 to 0.76, all above the 0.50 threshold [51, 52]. These results indicate strong internal consistency and adequate convergent validity for the PROMIS anger, anxiety, and fatigue scales in this sample (see Table S4).

### 3.3. Longitudinal Measurement Invariance

Longitudinal measurement invariance was evaluated for anger, anxiety, and fatigue across the two assessment waves using a sequence of nested models (Table 2). Configural invariance was first established, followed by tests of metric, scalar, and strict invariance. Because absolute RMSEA values can be inflated in large samples and longitudinal models, invariance decisions were based primarily on changes in fit indices rather than their absolute values. Across successive models, changes in CFI were below 0.01 and changes in RMSEA were below 0.015, meeting commonly recommended criteria for longitudinal measurement invariance. These results indicate that the measurement properties of anger, anxiety, and fatigue were stable across time.

### 3.4. Longitudinal Sequential Predictive Relationships Among Anger, Anxiety, and Fatigue

The full baseline‐controlled structural equation model demonstrated acceptable fit to the data (*χ* ^2^ = 1370.73, df = 47, *p* < 0.001, *χ* ^2^/df = 29.164, CFI = 0.969, TLI = 0.956, RMSEA = 0.090, and SRMR = 0.098). Although the RMSEA value exceeded conventional cutoffs for close fit, such elevations are common in two‐wave autoregressive models with large samples and limited degrees of freedom. In contrast, CFI and TLI—indices less sensitive to these conditions—indicated adequate overall model fit.

**Figure 1:**
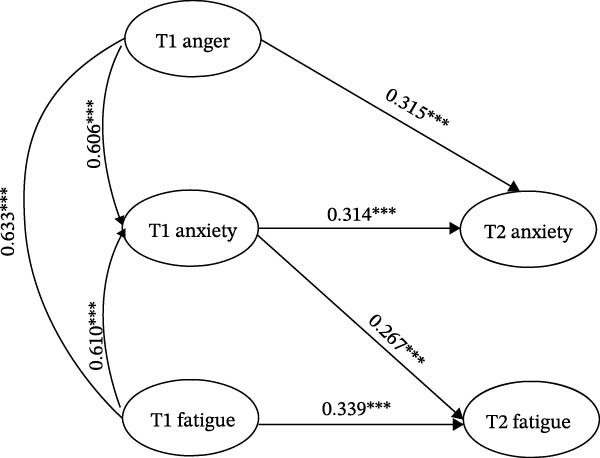
Sequential prediction model linking anger, anxiety, and fatigue (standardized coefficients). *Note:* This figure illustrates the two‐wave sequential prediction model examining whether anger assessed at baseline (T1) predicts subsequent anxiety at follow‐up (T2) and whether baseline anxiety predicts later fatigue. The model includes autoregressive paths for anxiety and fatigue; anger was modeled only at T1 because it served as the initial predictor in the sequential framework. All coefficients shown are standardized estimates. Abbreviations: T1 = baseline assessment; T2 = follow‐up assessment 6 months later.  ^∗∗∗^ *p* < 0.001.

Consistent with the hypothesized temporal sequence, T1 anger significantly predicted increases in T2 anxiety after controlling for baseline anxiety (*β* = 0.315, 95% CI [0.264, 0.368], *p* < 0.001). The autoregressive path from T1 anxiety to T2 anxiety was also significant (*β* = 0.314, 95% CI [0.267, 0.362], *p* < 0.001), indicating moderate temporal stability. In turn, T1 anxiety significantly predicted higher levels of T2 fatigue after controlling for baseline fatigue (*β* = 0.267, 95% CI [0.225, 0.310], *p* < 0.001), and the autoregressive path from T1 fatigue to T2 fatigue was likewise significant (*β* = 0.339, 95% CI [0.296, 0.382], *p* < 0.001). Baseline correlations among T1 anger, T1 anxiety, and T1 fatigue were all positive and statistically significant. All standardized path coefficients and confidence intervals are reported in Table 5, and the corresponding path diagram is shown in Figure 1.

**Table 5:** Primary sequential prediction model (*N* = 3475).

Path	*β* (95% CI)
T1 anger → T2 anxiety	0.315 ^∗∗∗^ [0.264, 0.368]
T1 anxiety → T2 fatigue	0.267 ^∗∗∗^ [0.225, 0.310]
T1 anxiety → T2 anxiety	0.314 ^∗∗∗^ [0.267, 0.362]
T1 fatigue → T2 fatigue	0.339 ^∗∗∗^ [0.296, 0.382]
Correlations (T1 anger, T1 anxiety)	0.606 ^∗∗∗^ [0.575, 0.640]
Correlations (T1 anxiety, T1 fatigue)	0.610 ^∗∗∗^ [0.583, 0.636]
Correlations (T1 anger, T1 fatigue)	0.633 ^∗∗∗^ [0.600, 0.664]

*Note*. Standardized coefficients are shown. All paths control for baseline levels of the outcome variable. Model fit: *χ* ^2^ = 1370.73, df = 47, *p* < 0.001, *χ* ^2^/df = 29.164, CFI = 0.969, TLI = 0.956, RMSEA = 0.090, SRMR = 0.098.  ^∗∗∗^ *p* < 0.001.

As supplementary descriptive information, paired‐sample *t*‐tests were conducted to compare mean levels of anger, anxiety, and fatigue between T1 and T2. These analyses were intended solely to characterize overall mean‐level changes over time and were not used to test the primary hypotheses, which focused on longitudinal predictive pathways within the SEM framework. Results indicated modest but statistically significant mean‐level changes across time for the study variables. Detailed statistics are reported in Table S1.

### 3.5. Supplementary Analyses: Uncontrolled Longitudinal Paths and Auxiliary Direct Effects

To assess the robustness of the primary findings and illustrate the impact of baseline autoregressive controls, supplementary analyses were conducted. First, uncontrolled longitudinal models without baseline adjustment yielded larger effect sizes for the T1 anger → T2 anxiety path (*β* = 0.487, *p* < 0.001) and the T1 anxiety → T2 fatigue path (*β* = 0.609, *p* < 0.001), indicating that controlling for baseline levels produces more conservative and temporally interpretable estimates. Second, simplified auxiliary structural models focusing on individual longitudinal associations (anger–anxiety, anxiety–fatigue, and anger–fatigue) demonstrated acceptable to excellent fit and results consistent with the primary sequential prediction model. Detailed results are provided in Tables S1–S3 and Figure S1.

### 3.6. Supplementary Gender‐Moderation Analyses

Supplementary regression‐based analyses were conducted to examine the role of gender in the longitudinal associations. Gender was first included as a covariate in the regression models. Results indicated that gender was modestly associated with anxiety at follow‐up (*β* = 0.04, *p* = 0.007), whereas its association with fatigue was not significant.

To further test whether the longitudinal pathways differed by gender, interaction terms between each T1 predictor and gender were entered into the models. A small but statistically significant interaction was observed for the T1 anger × gender term predicting T2 anxiety (*β* = 0.121, *p* < 0.01), accounting for a very limited increase in explained variance (Δ*R* ^2^ = 0.008). In contrast, the interaction between T1 anxiety and gender predicting T2 fatigue was not significant (*β* = 0.20, *p* = 0.311).

Overall, these results indicate that gender showed a small main effect on anxiety and a minimal moderating effect on the anger–anxiety pathway, while the anxiety–fatigue association did not differ by gender.

## 4. Discussion

This longitudinal study of 3475 university students investigated the temporal ordering among anger, anxiety, and fatigue. The findings consistently supported this sequential prediction pattern: anger at baseline forecasted heightened anxiety 6 months later, and heightened anxiety at baseline forecasted increased fatigue at follow‐up. Taken together, these results provide longitudinal support for a sequential prediction framework in which anger functions as an upstream emotional trigger, anxiety as an intermediate state, and fatigue as a downstream outcome among university students.

Consistent with prior research [18, 20, 22], the present results confirmed a robust prospective association between anger and fatigue. Students who reported higher anger at T1 were more likely to experience elevated fatigue at T2. This extends previous cross‐sectional findings by demonstrating a temporal relationship. From a self‐regulatory perspective, anger is a high‐arousal negative emotion that requires effortful control. Repeated attempts to inhibit, suppress, or modulate anger may draw upon limited self‐regulatory resources, thereby increasing vulnerability to subsequent fatigue [12]. In a university context characterized by academic demands, interpersonal stressors, and sleep disturbance, such self‐regulatory depletion may be especially salient. Thus, the current study strengthens the ecological validity of previous findings by demonstrating that anger prospectively predicts fatigue in everyday student life.

We also found that anxiety was strongly associated with fatigue across time, consistent with prior evidence from both clinical and community samples [24–26]. Extending this work to a nonclinical university population, our longitudinal findings show that students with higher anxiety at baseline were more likely to experience increased fatigue 6 months later. Clark and Watson’s [53] tripartite model provides a mechanistic explanation: anxiety is characterized by sustained physiological hyperarousal, which can disturb sleep architecture, impair metabolic restoration, and elevate subjective fatigue. Empirical work in adolescents [54] also links anxiety to somatic symptoms, supporting this physiological pathway. Our results align with this framework, suggesting that anxiety contributes to cumulative energy depletion among university students. Theoretically, this underscores the role of anxiety as a central mechanism through which negative affective states may translate into sustained fatigue in young adults.

The sequential analyses further indicated that anger precedes increases in anxiety over time. This is consistent with theoretical perspectives proposing that anger, when poorly regulated, disrupts interpersonal functioning and increases susceptibility to stress and threat sensitivity, thereby fostering heightened anxiety [55, 56]. Previous studies in older adults [39] also found anger to be a significant predictor of anxiety.

Given that anxiety is a well‐established risk factor for fatigue and CFS [26, 31], the longitudinal sequence observed in the present study—where anger prospectively predicts anxiety, and anxiety in turn forecasts subsequent fatigue—suggests a dynamic escalation process among negative emotional states in university students. In academic contexts characterized by high evaluative pressure, disrupted sleep routines, and irregular lifestyles [57, 58], heightened anger may amplify interpersonal strain and emotional arousal, thereby increasing vulnerability to anxiety [59] and, over time, contributing to fatigue‐related outcomes [60]. Extending prior cross‐sectional research, the present two‐wave design provides longitudinal evidence supporting this temporal ordering among anger, anxiety, and fatigue.

By explicitly modeling sequential predictive paths across two time points while controlling for baseline levels, the present study advances a process‐oriented account of emotional functioning that is consistent with resource depletion and self‐regulatory perspectives. Although a two‐wave design does not permit formal tests of longitudinal mediation, the observed pattern delineates a theoretically coherent temporal cascade from anger to anxiety to fatigue. In this respect, the findings offer empirically grounded guidance for future multiwave studies by clarifying the most defensible temporal ordering among these constructs, thereby informing and constraining subsequent tests of longitudinal mediation mechanisms.

Another contribution of this study lies in the simultaneous examination of anger and anxiety within a unified longitudinal framework. Previous research has predominantly focused on trait anger or trait anxiety as predictors of fatigue [61]. To avoid conceptual ambiguity between state and trait constructs, we conceptualized PROMIS anger and anxiety scores as indicators of state‐like emotional functioning, reflecting participants’ proximal emotional conditions over the past 7 days rather than enduring personality traits or momentary fluctuations. This intermediate conceptualization aligns with contemporary longitudinal research practices, in which short recall–based affective measures are used as time‐anchored predictors of subsequent psychological change. The present findings showed that these state‐like emotional conditions prospectively predicted later fatigue, suggesting that short‐term emotional dysregulation at baseline may initiate longer‐term cognitive and physiological processes that culminate in fatigue months later. From a theoretical standpoint, this supports the utility of proximal emotional indicators in models of fatigue development.

Despite its contributions, this study has several limitations that warrant consideration. First, although the longitudinal design allows for the examination of temporally ordered predictive relationships, it does not support strong causal inference [62, 63]. As emphasized by Cole and Maxwell [63], two‐time‐point data can establish temporal precedence but cannot formally test longitudinal mediation involving simultaneous estimation of direct and indirect effects. Accordingly, the present analyses were intentionally framed to focus on sequential prediction (anger → anxiety; anxiety → fatigue) rather than causal mediation. While this approach strengthens confidence in the temporal ordering of associations, alternative explanations—including reverse pathways or unmeasured third variables—cannot be fully ruled out. Future studies employing three or more waves will be necessary to formally evaluate longitudinal mediation and disentangle direct and indirect effects.

Second, the sample was drawn from a single university, which may limit the external validity of the findings. Institutional and regional characteristics of this setting may not fully represent the broader population of Chinese university students. Although the large sample size strengthens internal validity, caution is warranted when generalizing the observed anger–anxiety–fatigue sequence. Future research using multisite or nationally representative samples is needed to assess the robustness of these findings across diverse educational and cultural contexts.

Third, the 6‐month interval between assessments may not fully capture short‐term fluctuations in anger or acute fatigue episodes. Although this interval was selected to allow sufficient temporal separation for theoretically relevant processes (e.g., rumination, interpersonal strain, and hyperarousal) to unfold, future studies incorporating multiple waves with varying intervals could help disentangle short‐term dynamics from longer‐term emotional trajectories.

Fourth, sleep quality—widely recognized as a key factor associated with both anxiety and fatigue—was not assessed in the present study. As a result, we were unable to evaluate whether sleep disruption may partially confound or intervene in the observed longitudinal associations. Prior research indicates that anger and anxiety can impair sleep through heightened cognitive and physiological arousal [64, 65], while sleep disturbance is a well‐established contributor to subsequent fatigue [5, 66, 67]. Because sleep‐related variables were not included in the dataset, the present findings should be interpreted as reflecting a temporal pattern among emotional states rather than evidence for a sleep‐related mechanism. Future longitudinal studies incorporating validated sleep measures or objective sleep assessments will be essential to determine whether the identified anger–anxiety–fatigue sequence remains robust after accounting for sleep‐related processes.

Fifth, the sample was predominantly female (77.3%), which may limit generalizability given documented gender differences in internalizing symptoms among university students. Although prior research indicates that female students tend to report higher levels of anxiety, stress, and fatigue‐related outcomes [68–71], supplementary analyses in the present study suggested that gender did not meaningfully alter the core longitudinal associations. Specifically, only minimal gender‐related variability was observed for the anger → anxiety pathway, and no moderation was detected for the anxiety → fatigue pathway. These findings suggest that the temporal cascade from anger to anxiety to fatigue is broadly comparable across genders, although future studies with more balanced gender distributions are needed to more definitively examine gender‐specific mechanisms and boundary conditions.

## 5. Conclusion

This study demonstrated a clear sequential pattern among the three emotional states: baseline anger predicted later increases in anxiety, which in turn predicted subsequent increases in fatigue. Rather than interpreting this pattern as a formal mediation effect—which cannot be conclusively tested in a two‐wave design—we conceptualize it as a temporal cascade that reflects how short‐term emotional conditions may initiate longer‐term psychological processes contributing to fatigue. These findings refine existing affective‐process models by providing longitudinal evidence that dysregulated anger can function as an upstream emotional trigger that sets in motion anxiety‐related cognitive and physiological activation, ultimately accumulating into fatigue. The results highlight practical opportunities for early prevention within university mental health systems. Routine screening that incorporates brief assessments of anger and anxiety may help identify students at elevated risk for downstream fatigue. Interventions that strengthen adaptive emotion regulation—such as cognitive reappraisal training, mindfulness‐based strategies, or skills for managing anger‐eliciting situations—may reduce subsequent anxiety activation and mitigate longer‐term fatigue. However, these applications remain provisional and should be evaluated through controlled longitudinal or intervention studies to determine their efficacy in altering the temporal cascade identified in this study. Future studies should employ multiwave and shorter‐interval designs to capture dynamic emotional fluctuations more precisely and to test whether modifying anger‐ or anxiety‐related processes can interrupt this temporal sequence.

## Author Contributions

All authors contributed to the study conception and design. Haoran Guo was responsible for the data collection and data analysis and wrote the manuscript. Xiaofu Liu developed the manuscript preparation and review. Rong Fu participated in data curation and methodology. Lulu Ding carried out data visualization and investigation. Yanan Nie and Lingling Wang participated in methodology reviewing and editing. Shegang Zhou is considered the corresponding author and the chief investigator and is responsible for data analysis and manuscript supervision.

## Funding

This research was supported by the Henan Provincial Colleges Mental Health Education Famous Teacher Studio Project (Project Number 2022100003).

## Ethics Statement

All procedures performed in studies involving human participants were in accordance with the ethical standards of the institutional and/or national research committee. The Ethics Committee of the Institute of Education, Henan Normal University, approved the protocol of this study (Project Identification Code 2022100003). The questionnaires were systematically distributed and collected by qualified psychologists.

## Consent

All participants provided verbal informed consent prior to their participation in the study. Participants were informed that they were under no obligation to participate and that they could withdraw at any time from the study without consequences.

## Conflicts of Interest

The authors declare no conflicts of interest.

## Supporting Information

Additional supporting information can be found online in the Supporting Information section.

## Supporting information


**Supporting Information** **Table S1:** Descriptive statistics and paired‐samples *t*‐tests comparing T1 and T2 anger, anxiety, and fatigue (*N* = 3475). **Table S2:** Sequential prediction model of anger, anxiety, and fatigue (*N* = 3475). **Table S3:** Structural equation model testing the direct effect of T1 anger on T2 fatigue (*N* = 3475). **Table S4:** Composite reliability (CR) and average variance extracted (AVE). **Figure S1:** SEM path diagram showing the direct T1 anger → T2 fatigue effect. *Note:* Supplementary SEM depicting the direct path from T1 anger to T2 fatigue (controlling for T1 fatigue). Standardized coefficients are displayed. Abbreviations: T1 = baseline assessment; T2 = follow‐up assessment six months later.  ^∗∗∗^ *p* < 0.001.

## Data Availability

The data that support the findings of this study are available upon request from the corresponding author. The data are not publicly available due to privacy or ethical restrictions.
