# Supplementary material for: Anger and Anxiety as Sequential Predictors of Fatigue: A Two‐Wave Longitudinal Study
Source: Depress Anxiety. 2026 Feb 23;2026:8772749. doi: 10.1155/da/8772749 (PMC12927898; doi:10.1155/da/8772749)
Supplement: Supplementary file 1 — Supporting Information Table S1: Descriptive statistics and paired‐samples t‐tests comparing T1 and T2 anger, anxiety, and fatigue (N = 3475). Table S2: Sequential prediction model of anger, anxiety, and fatigue (N = 3475). Table S3: Structural equation model testing the direct effect of T1 anger on T2 fatigue (N = 3475). Table S4: Composite reliability (CR) and average variance extracted (AVE). Figure S1: SEM path diagram showing the direct T1 anger → T2 fatigue effect. Note: Supplementary SEM depicting the direct path from T1 anger to T2 fatigue (controlling for T1 fatigue). Standardized coefficients are displayed. Abbreviations: T1 = baseline assessment; T2 = follow‐up assessment six months later. ∗∗∗ p < 0.001. [file DA-2026-8772749-s001.docx]

Supplementary Table S1: Descriptive statistics and paired-samples t-tests comparing T1 and T2 anger, anxiety, and fatigue (*N* = 3,475)

| Variable | Time | M | SD | Mean difference (T1-T2) | 95% CI of difference | *t* | *p* |
| --- | --- | --- | --- | --- | --- | --- | --- |
| Anger | T1 | 10.66 | 3.62 | 0.73 | [0.61,0.86] | 111.12^***^ | <0.001 |
| Anger | T2 | 9.92 | 3.78 |  |  |  |  |
| Anxiety | T1 | 16.49 | 6.19 | 0.90 | [0.69,1.11] | 8.47^***^ | <0.001 |
| Anxiety | T2 | 15.59 | 6.02 |  |  |  |  |
| Fatigue | T1 | 21.81 | 7.69 | 1.63 | [1.37,1.90] | 12.05^***^ | <0.001 |
| Fatigue | T2 | 20.18 | 7.91 |  |  |  |  |

*Note.* Means and standard deviations are based on raw scale scores. Mean differences are computed as T1 - T2; positive values indicate lower levels at T2. All paired-samples t-tests are significant at *p* < 0.001.

Supplementary Table S2: Sequential prediction model of anger, anxiety, and fatigue (*N*=3475).

| Path | Model 1  (No controls) | Model 2  (No controls) | Model 3  (Controls included |
| --- | --- | --- | --- |
| T1 anger → T2 anxiety | 0.487^***^  95% CI [0.451,0.522] |  | 0.315^***^  95% CI [0.264,0.368] (Control: T1 anxiety) |
| T1 anxiety → T2 fatigue |  | 0.609^***^  95% CI [0.582, 0.635] | 0.267^***^  95% CI [0.225,0.310] (Control: T1 anxiety) |
| T1 anxiety → T2 anxiety |  |  | 0.314^***^  95% CI [0.267,0.362] |
| T1 fatigue → T2 fatigue |  |  | 0.339^***^  95% CI [0.296,0.382] |

Model Fit: Model 1 (T1 anger → T2 anxiety; no controls): χ²(4) = 69.415, *p* < 0.001, χ²/df = 17.354, CFI = 0.995, TLI = 0.987, RMSEA = 0.069, SRMR = 0.005.

Model 2 (T1 anxiety → T2 fatigue; no controls): χ²(4) = 13.619, *p* <0 .001, χ²/df = 3.405, CFI = 0.999, TLI = 0.999, RMSEA = 0.026, SRMR = 0.004.

Model 3 (Full sequential predictive model with controls): χ²(47) = 1370.73, *p* < 0.001, χ²/df = 29.164, CFI = 0.969, TLI = 0.956, RMSEA = 0.090, SRMR = 0.098.

*Note:* Table S1 presents the primary sequential prediction model, which specifies the temporal paths from T1 anger to T2 anxiety and from T1 anxiety to T2 fatigue while controlling for baseline levels of each construct. All coefficients are standardized. T1 = baseline assessment; T2 = follow-up assessment six months later.

Abbreviations: ^***^*p* < 0.001.

Supplementary Table S3: Structural equation model testing the direct effect of T1 anger on T2 fatigue (*N*=3475).

| Path | Model 4 |
| --- | --- |
| T1 anger → T2 fatigue | *β* = 0.396^***^, 95% CI [0.316,0.477] |
| T1 fatigue → T2 fatigue | *β* = 0.355^***^, 95% CI [0.303,0.407] |
| Correlations (T1 anxiety, T1 fatigue) | *β* = 0.625^***^, 95% CI [0.587,0.651] |

Model Fit: Model 4 (Direct effect model: T1 anger → T2 fatigue, controlling for T1 fatigue): χ²(6) = 97.405, *p* < 0.001, χ²/df = 16.234, CFI = 0.994, TLI = 0.986, RMSEA = 0.066, SRMR = 0.005.

*Note:* This supplementary SEM estimates the direct longitudinal path from T1 anger to T2 fatigue while statistically controlling for baseline fatigue. All coefficients are standardized. T1 = baseline assessment; T2 = follow-up assessment six months later.

Abbreviations: ^***^*p* < 0.001.


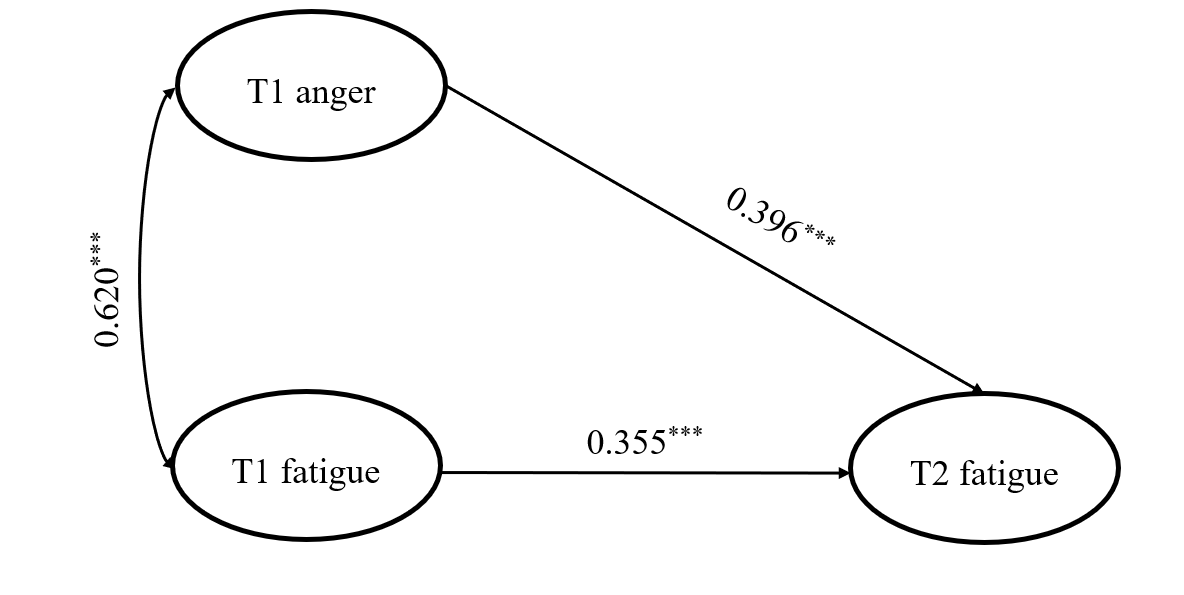


Supplementary Figure 1: SEM path diagram showing the direct T1 anger → T2 fatigue effect

*Note:* Supplementary SEM depicting the direct path from T1 anger to T2 fatigue (controlling for T1 fatigue). Standardized coefficients are displayed.

Abbreviations: T1 = baseline assessment; T2 = follow-up assessment six months later. ^***^*p* < 0.001.

Supplementary Table S4: Composite reliability (CR) and average variance extracted (AVE)

| Construct | Time | CR | AVE |
| --- | --- | --- | --- |
| Anger | T1 | 0.89 | 0.63 |
| Anger | T2 | 0.92 | 0.69 |
| Anxiety | T1 | 0.95 | 0.72 |
| Anxiety | T2 | 0.95 | 0.71 |
| Fatigue | T1 | 0.96 | 0.70 |
| Fatigue | T2 | 0.97 | 0.76 |

*Note*. CR = composite reliability; AVE = average variance extracted. Values > 0.70 for CR indicate good reliability; values > 0.50 for AVE indicate adequate convergent validity. Estimates are based on standardized factor loadings from confirmatory factor analyses.
